# *Cycas micronesica* Stem Carbohydrates Decline Following Leaf and Male Cone Growth Events

**DOI:** 10.3390/plants9040517

**Published:** 2020-04-17

**Authors:** Thomas E. Marler, Gil N. Cruz

**Affiliations:** College of Natural and Applied Sciences, University of Guam, Mangilao, GU 96923, USA; gilcruz@triton.uog.edu

**Keywords:** conservation physiology, starch, source–sink relations, sucrose

## Abstract

The growth of synchronized leaf flushes or male cones on *Cycas* trees is an ephemeral event, and non-structural carbohydrates (NSCs) are likely deployed from stem and root storage tissues to support their construction. The relationships among various stem NSCs and these rapid growth events have not been studied to date. Monosaccharides, disaccharides, and starch were quantified in *Cycas micronesica* stem tissue prior to and immediately after the growth of leaf flushes or male cones to determine the influences on the concentration of these carbohydrates. The pre-existing leaves were removed from half of the plants to determine if the elimination of this carbon source would influence the NSC behaviors. Starch and sucrose dominated the NSC profiles, and these two NSCs declined following cone or new leaf growth. Removal of pre-existing leaves generated a greater decline in starch and sucrose for cone growth, and a greater decline in sucrose, but not starch following new leaf growth than in control trees with no leaf removal. The initial differences in starch and sucrose among cortex, vascular, and pith tissues disappeared as the concentrations declined in all three tissue categories to reach similar post-growth concentrations among the stem tissue categories. The fructose, glucose, and maltose behaviors were not consistent, and their concentrations were low such that their influence on the total NSC behaviors was minimal. These results provided indirect evidence that stem NSCs were mobilized to support ephemeral male cone and new leaf growth for this arborescent cycad. Growth of female strobili is slow and lengthy, so we did not include female trees in this study. The contributions of stem NSCs to female strobili growth remain to be studied with alternative methods.

## 1. Introduction

Cycads are long-lived perennial gymnosperm plants that exhibit discontinuous primary growth characterized by long periods of apparent quiescence between ephemeral bursts of leaf or reproductive structure growth from stem apices [1]. The cycad pachycaulous stems are manoxylic and lack the bifacial secondary cambium present in lignophyte tree species for radial growth [1,2]. Persistent pith and cortex are separated radially by concentric vascular cylinders that contain copious parenchyma tissue [3,4,5]. Living parenchyma tissue persists across the entire radial area of the cycad stem. As a result, abundant non-structural carbohydrates are stored in cycad stems. Carbohydrate resources using contemporary analytical approaches have been quantified for *Cycas micronesica* K.D. Hill [6,7], *Cycas revoluta* Thunb. [8], and *Zamia muricata* Willd. [9].

**Figure 1:**
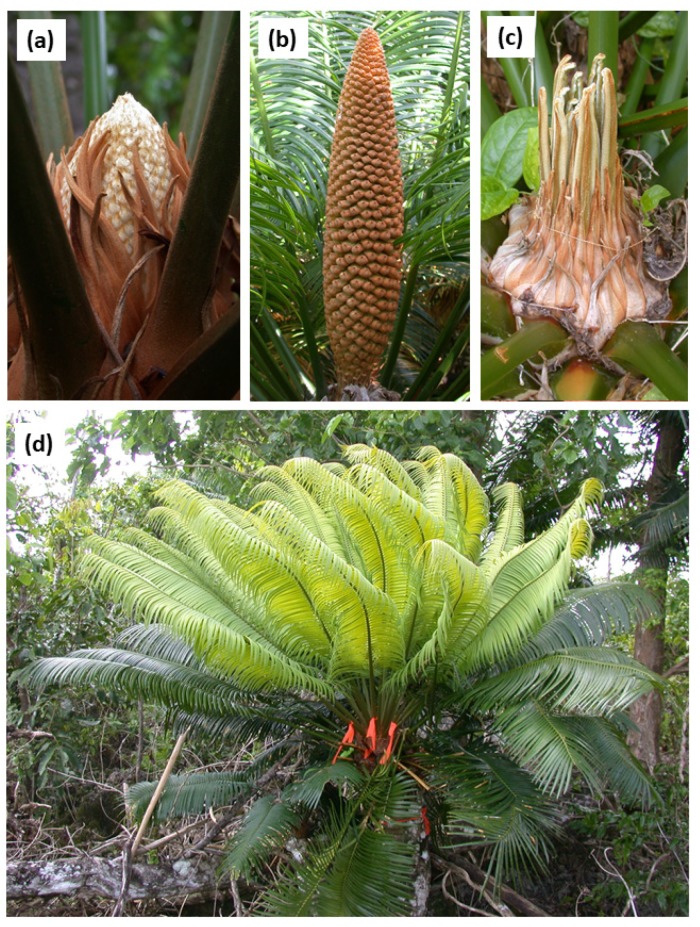
Male cone or synchronized leaf growth occurs from the apex of *Cycas micronesica* stems. (**a**) Male cone two days after emergence. (**b**) Mature male cone with eight weeks of growth. (**c**) Leaf growth five days after emergence, showing circinate leaflets. (**d**) Fully expanded leaf flush with four weeks of growth.

The study of phenology of cycad species within in situ localities has been deficient. While studying *C. micronesica* phenology, we determined that leaf flushes emerge and complete expansion in about one month, while male cones emerge and complete expansion in about two months (Figure 1) [10]. These ephemeral growth events may occur during any month of the year for Guam localities, but are more pronounced in April for male cones and August for leaf flushes [11].

The rapid pulse of growth of new synchronized leaves or male cones offers a model system that lends itself to the study of concomitant sink activity. Female reproductive structures also add expensive construction events to a *Cycas* tree. Nevertheless, the sporophylls, ovules, and developing seeds of the *C. micronesica* megastrobilus are slowly developing structures that require years to mature [10]. Moreover, the addition of a new leaf flush subsequent to the addition of a megastrobilus complicates the model system with a carbon source that is younger than the megastrobilus. In contrast, a new synchronized leaf flush or male cone reaches mature size rapidly and without the addition of a younger carbon source, forcing the growth to be supported by current photosynthates of pre-existing leaves and non-structural carbohydrates (NSCs) stored in stems and roots.

*Cycas micronesica* leaves are 150–180 cm in length [12] and 25–30 leaves may occur in a single flush for mature trees. The construction of so much new tissue within one month suggests that an abundance of construction resources is likely to be needed within a short time period. Although male cones require twice as much time to reach mature size, these structures are sizeable and commonly reach 50 cm in height and 10 cm in diameter [12]. Moreover, thermogenic activity, which requires non-structural resources as substrates, is a crucial component of *C. micronesica* male cone behavior [13,14]. We predicted that a decline in *C. micronesica* stem NSC concentrations would occur in response to the sink demands of the rapid expansion of these two organs, with a greater reduction in the NSC resources of the stem by construction of the male cone rather than the leaf flush construction.

## 2. Materials and Methods

### 2.1. Site Description

The experimental site was a plot in a native forest on the east coast of Guam in which all *C. micronesica* trees were protected from the lethal *Aulacaspis yasumatsui* Takagi with granular imidacloprid applications since 2007. The plot was positioned at 20–60 m above sea level, aspect was 120–150°, and mature plant density was 1050 stems per ha. More details for the site are contained elsewhere [7,15,16]. The edaphic substrates at this site were coralline soils formed in slope alluvium, loess, and residuum overlying limestone (clayey-skeletal, gibbsitic, nonacid, isohyperthermic Lithic Ustorthents) [17].

*Cycas micronesica* phenology in this and other localities on Guam had been observed for several years. Although leaf or cone emergence may occur at any time throughout the year, the most synchronized population-level male cone emergence occurred in April, and the most synchronized population-level leaf flush emergence occurred in August. These two months were selected for the two sampling periods in 2012 when the plants had been protected from *A. yasumatsui* infestations for five years. The protected trees were vigorous in appearance and possessed robust crowns of 50–70 healthy leaves per tree. Most of the mature trees in the plot had been identified as male or female, and the experimental trees for this study were restricted to male trees. These methods enabled a more accurate comparison between the influences of leaf flushes and male cone growth, as the sex of cycad plants has been shown to affect organ chemistry [18,19].

### 2.2. Field Methods

The male tree population within the protected plot was observed twice weekly in April 2012 for newly emerging cones until six pairs of trees with synchronized dates of cone emergence were identified (Table 1). The same population was observed twice per week in August 2012 for new leaf flushes until six pairs of trees with synchronized dates of leaf emergence were identified. The observed trees during this reconnaissance were restricted to heights of 2.0–2.5 m to remove any inconsistencies owing to allometric relations. Moreover, any trees with a cone as the antecedent growth event were not included to ensure that all 12 trees were individuals with two successive leaf flushes prior to the experimental period. The pairs were created by proximity within the plot and synchrony of organ emergence. The two trees for each pair were separated by no more than 30 m. For each pair of synchronized trees, one of the trees was randomly selected for defoliation to force the organ construction to rely on stored NSCs for construction.

Total stem height and stem diameter at the base were measured (Table 1). Field methods for excision of stem tissue were patterned after Marler and Shaw [20]. A 2.54 cm hole saw was installed in a hand drill and a core was extracted at 30 cm below the oldest leaf petiole. This position was 40–50 cm below the stem apex. Each core extended through the cortex and vascular cylinders to a depth that extracted 1 cm of pith tissue. Stem diameter at the height of the core was measured. The field tissue samples were handled as described in [7] and transported to a University of Guam laboratory, where each core was separated into cortex, vascular, and pith categories. The diameter of the cortex and vascular tissue was measured directly. The diameter of pith was calculated by subtracting the cortex and vascular tissue diameter from the total stem diameter by assuming the cortex and vascular tissue diameter on the opposite side of the core was in symmetry with that on the side of the core.

For each tree, one core was extracted prior to the cone or leaf flush. For the cone growth study, this was 4–25 April 2012, and for the leaf growth study, this was 2–26 August 2012. A second core was extracted from a different radial orientation immediately after the culmination of growth, after one month for the leaf flush trees, and immediately after pollen dispersal for the male cone trees. For the cone growth study, this was carried out between 10 and 30 June 2012, and for the leaf growth study, this occurred between 3 and 25 September 2012.

The hole created by each core was protected on the same date by filling the hole with expanding insulation foam (Great Stuff^TM^, Dow Chemical Company, Wilmington, IL, USA) [21]. The foam was allowed to cure for one day, and then was trimmed to be flush with the stem surface. Tree wound sealant was applied over the surface of the foam and the 3 cm surrounding the wound. Verification that no secondary damage occurred to the intact stem tissues following the core extractions was achieved by monitoring the trees for three years, when the imidacloprid applications were terminated owing to loss of funding.

### 2.3. Tissue and Data Analysis

The frozen tissue was lyophilized and milled to pass through a 20 mesh screen. NSC was quantified as described in [6,7,8]. Soluble sugar extraction was conducted using hot-water extraction with acetonitrile (80 °C) [22]. The concentrations of four free sugars (the hexoses fructose and glucose, and the disaccharides sucrose and maltose) were determined by HPLC-RI (Thermo Scientific RI-150, AS3000 autosampler, P2000 pump, Waltham, MA, USA). Starch was quantified following hydrolyzation by amyloglucosidase to glucose [23].

The MIXED procedure (Proc MIXED, SAS Institute, Cary, NC, USA) was employed for the three-way analysis of variance in a split-split-plot design. The whole plot treatment was defoliation treatment, the sub-plot treatment was date, and the sub-sub-plot treatment was tissue category. The response variables were starch, the two disaccharides, the two monosaccharides, and total NSC as derived by the sum of the five measured NSCs. Male cone and new leaf data were analyzed separately because the two studies were conducted in different seasons.

## 3. Results

### 3.1. Male Cone Trees

**Figure 2:**
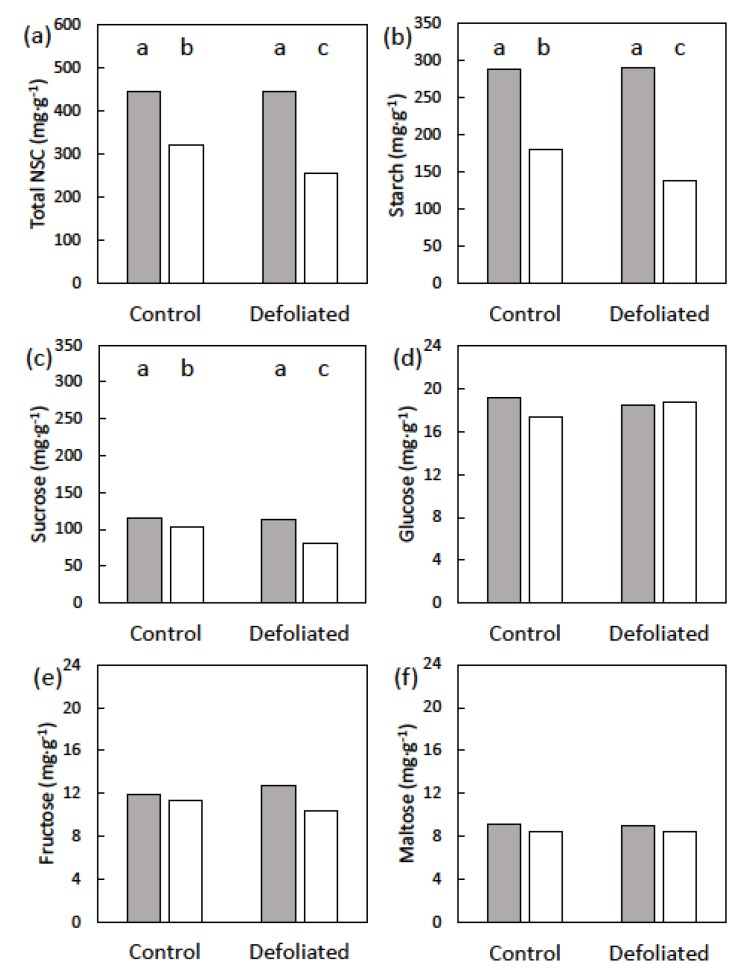
The non-structural carbohydrates (NSC) of *Cycas micronesica* stem tissues before (gray bars) and after (white bars) the construction of male cones, as influenced by defoliation prior to cone growth. (**a**) Total NSC; (**b**) starch; (**c**) sucrose (**d**) glucose; (**e**) fructose; and (**f**) maltose. Bars with same letters are not different for NSCs with significant time × treatment effects.

The 12 male cones were typical in appearance and size. At the pollen dispersal stage when maximum size occurred, the cones ranged from 36 to 44 cm in height for the control trees and from 35 to 45 cm in height for the defoliated trees. The stem carbohydrate concentrations ranked in the order starch > sucrose > glucose > fructose > maltose. Starch exhibited significant differences for the treatment × date interaction (*p* = 0.026). Compared with the initial concentrations, the defoliation treatment produced a 43% reduction in stem starch concentration compared with 38% in the control treatment (Figure 2). A similar pattern was observed with sucrose, with defoliated trees having a greater decline in sucrose than the control trees (29% versus 10% decline, respectively; *p* = 0.011). The treatment × date interaction was not significant for glucose, fructose, or maltose. Furthermore, the treatment main factor was not significant for any of these sugars. The date main factor was not significant for glucose or fructose, but was significant for maltose (*p* < 0.001). The post-cone maltose concentration was 7% below that of the pre-cone maltose concentration. Total NSC exhibited significant differences among the treatment × date interaction (*p* = 0.007), and patterns followed those of starch (Figure 2).

**Figure 3:**
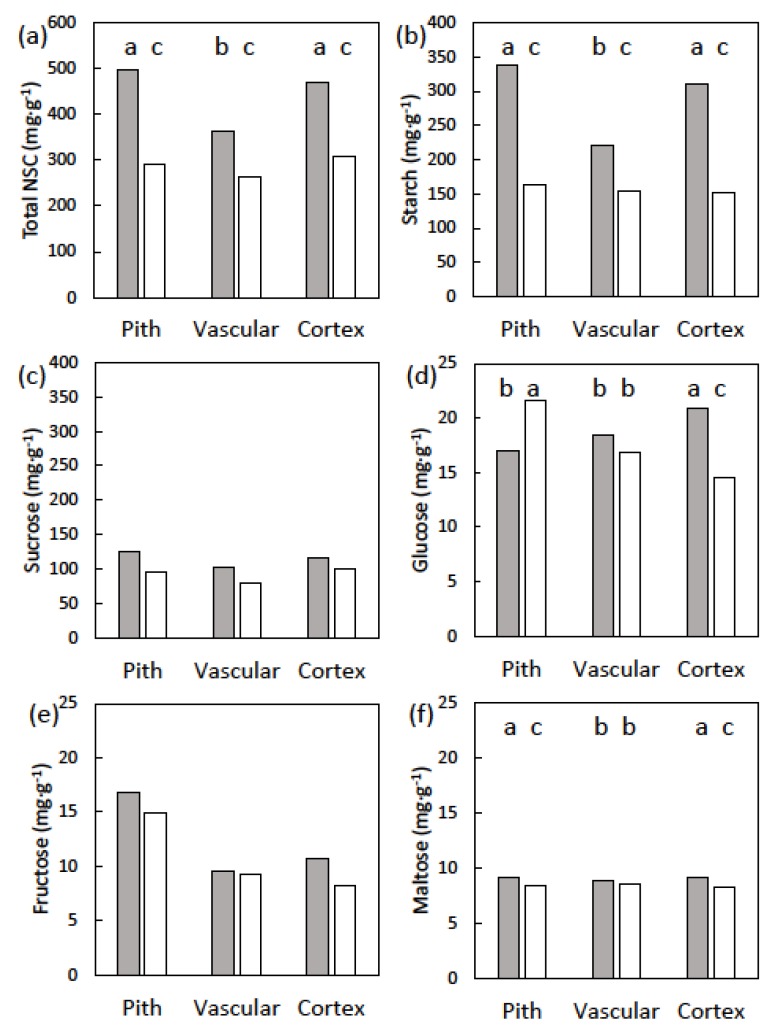
The non-structural carbohydrates (NSC) of *Cycas micronesica* stem tissues among three radial tissues before (gray bars) and after (white bars) the construction of male cones. Defoliated trees had leaves removed prior to emergence of cones. (**a**) Total NSC; (**b**) starch; (**c**) sucrose; (**d**) glucose; (**e**) fructose; and (**f**) maltose. Bars with same letters are not different for NSCs with significant time × tissue effects.

Stem starch concentration exhibited a date × tissue type interaction for these trees with male cones (*p* < 0.001). Stem starch concentration varied among cortex, vascular, and pith tissues prior to cone growth, but did not differ among the tissue categories following cone maturation (Figure 3). Stem sucrose concentration in vascular tissue was less than that in cortex or pith tissue prior to cone emergence and following cone maturation (*p* < 0.001). These similar patterns before and after the cone growth caused the date × tissue type interaction to be non-significant (*p* = 0.157). However, the date main factor was significant (*p* < 0.001), and stem sucrose after cone maturation was 80% of that prior to cone emergence (Figure 3). The pattern of glucose concentration before and after cone growth was unique (Figure 3). Stem glucose concentration declined in vascular and cortex tissue following cone maturation, but increased in pith tissue. Stem fructose concentration was not influenced by date (*p* = 0.414), or by the date × tissue interaction (*p* = 0.361). In contrast, stem fructose concentration varied among the tissue categories (*p* < 0.001). Fructose concentration was similar between pith and vascular tissues, and was 1.7-fold greater in cortex tissue (Figure 3). Stem maltose concentration exhibited a date × tissue type interaction (*p* = 0.015). Maltose concentration was similar for pith and cortex tissue before cone emergence, and declined similarly about 7% during cone maturation (Figure 3). Vascular tissue maltose concentration was less than that of cortex or pith tissue, and was not influence by date. Total NSC concentration in the stem tissues exhibited a date × tissue type interaction (*p* < 0.001). The patterns among tissue types and dates were similar to those of starch (Figure 3).

### 3.2. Leaf Flush Trees

**Figure 4:**
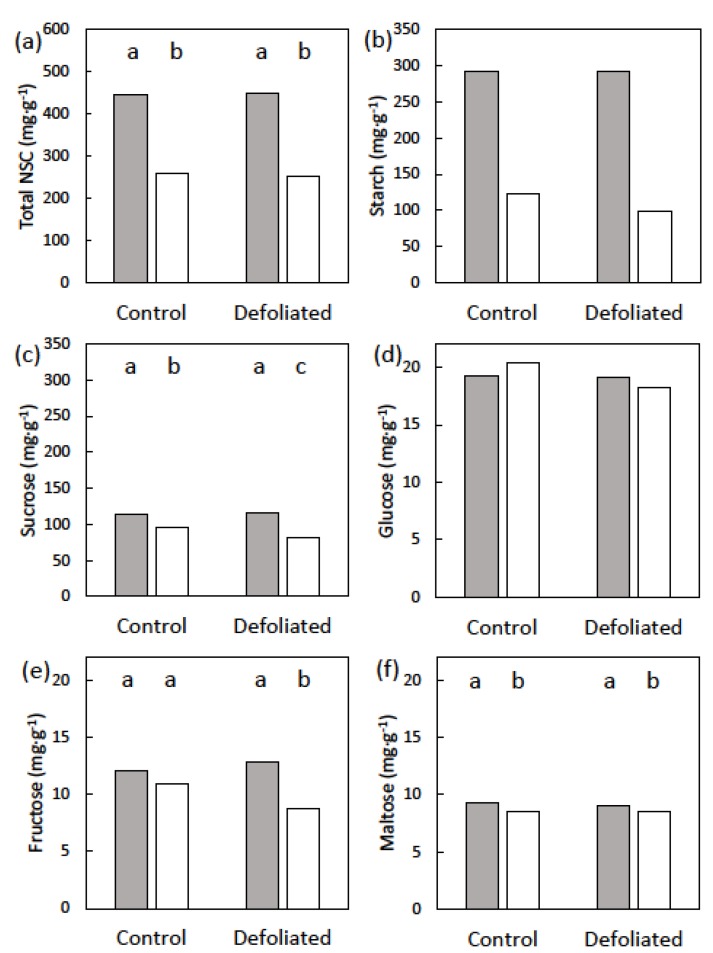
The non-structural carbohydrates (NSC) of *Cycas micronesica* stem tissues before (gray bars) and after (white bars) the construction of synchronized leaf flush, as influenced by defoliation prior to new leaf growth. (**a**) t=Total NSC; (**b**) starch; (**c**) sucrose (**d**) glucose; (**e**) fructose; and (**f**) maltose. Bars with same letters are not different for NSCs with significant time × treatment effects.

The numbers of leaves within the synchronized flushes were similar among the 12 trees that were used for the leaf flush measurements. There were 15–22 or 16–21 leaves per flush for the control or defoliated trees, respectively. Mature length of the new leaves was 166–182 cm for the control trees, and was reduced by pre-existing leaf removal to 145–163 cm for the defoliated trees. The pre-emergence concentrations of the NSCs among the three tissue types were similar to those for the male cone trees. Starch exhibited significant differences for the treatment × date interaction (*p* = 0.021). Stem starch concentration after leaf expansion was 58% or 66% below the concentration prior to leaf emergence in control trees or prior to leaf emergence in defoliated trees (Figure 4). Sucrose was the free sugar in highest concentration and showed significant differences in the treatment × date interaction (*p* = 0.037). Stem sucrose concentration after leaf expansion was 16% and 30% below that prior to leaf emergence in control trees and defoliated trees, respectively (Figure 4). Stem glucose concentration was not influenced by date (*p* = 0.822), treatment (*p* = 0.403), or the date × treatment interaction (*p* = 0.658). Stem fructose concentration exhibited significant differences among the treatment × date interaction (*p* = 0.004). Stem fructose concentration after leaf expansion was 10% and 33% below that prior to leaf emergence in in control and defoliated trees, respectively (Figure 4). Stem maltose concentration was not influenced by treatment (*p* = 0.307) or the date × treatment interaction (*p* = 0.383). However, the stem maltose concentration was influenced by date (*p* < 0.001). The maltose concentration after leaf expansion was 92% of that of the pre-growth maltose concentration (Figure 4). Total NSC patterns followed those of starch for dates and treatments (Figure 4). The before cone emergence and after cone maturation concentrations were similar for the two treatments, so the treatment × date interaction was not significant (*p* = 0.554). The date main factor was significant (*p* < 0.001), and stem NSC concentration after cone maturation was 43% below that of NSC concentration prior to cone emergence.

**Figure 5:**
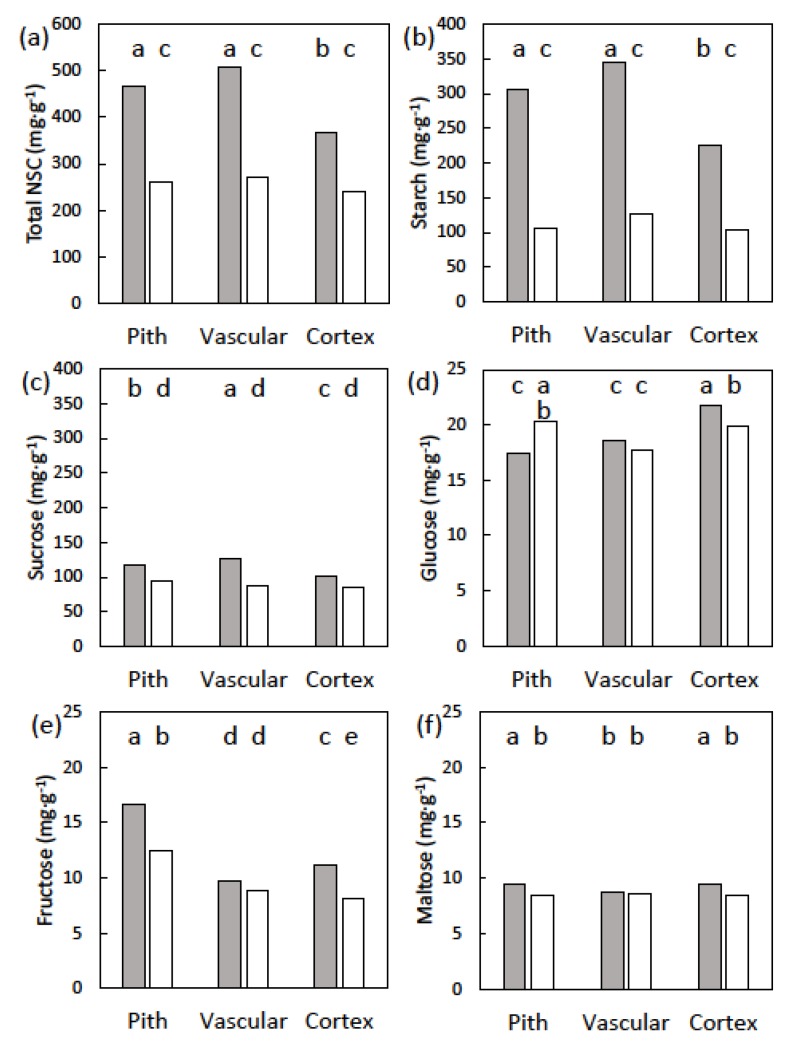
The non-structural carbohydrates (NSCs) of *Cycas micronesica* stem tissues among three radial tissues before (gray bars) and after (white bars) the construction of synchronized leaf flush. Defoliated trees had leaves removed prior to emergence of the new leaves. (**a**) Total NSC; (**b**) starch; (**c**) sucrose (**d**) glucose; (**e**) fructose; and (**f**) maltose. Bars with same letters are not different for NSCs with significant time × tissue effects.

Stem starch concentration exhibited a date × tissue type interaction for these trees exhibiting a leaf flush (*p* < 0.001). Stem starch concentration varied among cortex, vascular, and pith tissues prior to leaf growth, but did not differ among the tissue categories following leaf maturation (Figure 5). Stem sucrose concentration also exhibited a date × tissue type interaction for these trees exhibiting a leaf flush (*p* < 0.001). As with starch, stem sucrose concentrations varied among the organs prior to leaf emergence, but were reduced to similar concentrations after leaf expansion (Figure 5). Stem glucose concentration exhibited a date × tissue type interaction for these trees exhibiting a leaf flush (*p* < 0.001). Pith glucose concentration increased, vascular glucose concentration was unaffected, and cortex glucose concentration decreased during leaf expansion (Figure 5). Stem fructose concentration was influenced by the date × tissue interaction (*p* = 0.007). Fructose concentration decreased during leaf expansion for pith and cortex tissues, but was unaffected for vascular tissue (Figure 5). Stem maltose concentration exhibited date × tissue type interaction (*p* < 0.001). Maltose concentration was similar for pith and cortex tissue before leaf emergence, and declined a similar amount during cone maturation (Figure 5). Vascular tissue maltose concentration was less than that of cortex or pith tissue, and was not influenced by date. Total NSC concentration in the stem tissues exhibited a date × tissue type interaction (*p* < 0.001). The patterns among tissue types and dates were similar to those of starch (Figure 5).

## 4. Discussion

### 4.1. Stem NSC Behavior

Starch and disaccharides dominated the NSC relations in the *C. micronesica* stem tissue. The patterns of declines of these two NSCs in stem tissue were similar for rapid new leaf or male cone growth. The fructose, glucose, and maltose concentrations were minimal compared with starch and sucrose concentrations, and the behaviors in response to rapid new leaf or male cone growth were not consistent for these three sugars. These results generally confirmed our first prediction, and indirectly verified that the sink activity of rapid leaf and cone growth placed demands on stem NSCs. Two outcomes of our study were not consistent with the prediction that male cone growth would generate greater demands on stem NSCs than new leaf growth. First, the decline in NSC concentration following new leaf growth was greater than the decline following male cone growth. Second, the decline in stem starch during male cone growth was amplified by removing all pre-existing leaves, but the decline in stem starch during new leaf growth was not influenced by removing the pre-existing leaves. These results indirectly verified that new leaf growth placed more demands on labile stem NSCs than new cone growth, and that some of the sink demands for cone maturation were met from current photosynthates from subtending leaves.

The study of cycad stem NSC relations using contemporary analytical approaches is limited, but the few reports on *Cycas* plants have confirmed that these manoxylic stems contain copious NSC resources. Container-grown *C. revoluta* plants exhibited stem starch of ≈200 mg·g^−1^ and NSC of ≈380 mg·g^−1^ [8]. Container-grown *C. micronesica* plants exhibited stem starch of ≈129 mg·g^−1^ and NSC of ≈314 mg·g^−1^ [6]. In situ *C. micronesica* plants exhibited stem starch of ≈200 mg·g^−1^ and NSC of ≈370 mg·g^−1^ [7]. Our results from healthy male *C. micronesica* trees that were protected from herbivory with systemic insecticides exceeded these published values, with starch concentrations close to 300 mg·g^−1^ and NSC of more than 400 mg·g^−1^.

The sampling cores were restricted to a single axial location immediately below the living leaves. Therefore, our results are not useful for determining how the sink activity of new leaf flushes and male cones on *Cycas* stem apices influence root NSC resources and stem NSC resources at axial locations closer to the base of the stem. Our results also do not contribute to our need to understand the processes whereby stem NSC concentrations are replenished after the ephemeral declines owing to the temporary sink activity. Indeed, the costs and benefits of stem parenchyma abundance in trees are not well-understood [24]. In source–sink studies, the most direct approach for determining the fate of NSCs includes carbon pulse-chase labelling methods, but to our knowledge, these methods have never been applied to a cycad taxa. Despite the indirect nature of our study, the outcomes confirm that starch and sucrose were the primary constituents of the *C. micronesica* stem NSCs, and both of these NSCs declined during the ephemeral rapid expansion of leaves or male cones. Moreover, the initial concentrations were similar among the radial tissue categories and became even more similar following the sink activity.

The various outcomes from these manipulative studies enabled two further interpretations. First, cone size and leaf number per flush were not influenced by retention or removal of pre-existing leaves, providing indirect evidence that these traits were either fixed prior to organ emergence or that the labile stem resources alone determined the outcomes. Second, leaf length was reduced by removal of all pre-existing leaves, providing indirect evidence that concomitant contributions of leaf photosynthates from pre-existing leaves were required for newly added leaves to reach maximum size.

This case study builds on our earlier work within the developing sub-discipline of conservation physiology. For example, manual removal of male cones or consumption of male cone tissue by herbivores hastened subsequent leaf and cone production for *C. micronesica* trees [25]. The level of exposure to abiotic stressors between two habitats influenced reproductive effort of female *C. micronesica* trees [26]. These observations further support the assertions that the ephemeral bursts of organ expansion in this cycad species may be mediated by a resource trade-off in optimal-allocation models [27]. The cumulative knowledge about stem NSCs also conforms to our resource depletion model concerning the means by which *A. yasumatsui* infestations cause mortality of *Cycas* plants [8]. For healthy plants, reductions in stem NSCs during leaf construction are transient and the long period of apparent quiescence that follows every leaf flush characterizes a period of time during which the newly added leaves can pay the stem tissues back for their construction costs. However, during lethal *A. yasumatsui* infestations, the newly added leaves are damaged and killed before they have an opportunity to pay back those construction costs. Following repetitive leaf flush events, a plant reaches the point of no return as stem NSCs steadily decline over time under the *A. yasumatsui* pressures.

### 4.2. Traditional Knowledge

Our findings offer new insights to concepts that are woven into the traditional knowledge of many ethnic groups. Starch that is harvested from cycad organs has long been used for food or alcoholic beverages [1]. While seeds have been the primary source of cycad starch, harvesting of stems for human consumption has been reported for *Bowenia* Hook., *Cycas*, *Dioon* Lindl., *Encephalartos* Lehm., *Macrozamia* Miq., and *Zamia* L. species [28,29,30,31]. The traditional knowledge that supports the harvest and preparation protocols for cycad stem starch indicates that male stems yield more starch than female stems, that more than one year may be required for a female stem to recover starch levels following the initiation of a megastrobilus, that starch yields vary among localities of the same species, and that the best time for harvesting a stem for starch extraction is immediately prior to a leaf flush [19]. The behaviors of cycad stem starch provide an ideal example of the value of traditional knowledge for steering modern research agendas. For example, the contributions of stem NSCs to female strobili growth remain to be studied with alternative methods.

### 4.3. Future Directions

The differences between male cone construction and new leaf flush construction deserve further study, and several issues may be valuable for pursuing these efforts. First, the justification for predicting a greater cost of male cone growth was not just a presumed greater level of dry matter production for the cones, but was also because leaf expansion is supported by photosynthetic activity of the expanding leaves and, therefore, construction costs may be less dependent on stored stem resources than expanding cones. Although the timing of the sink–source transition is not known for construction of leaves for any *Cycas* species, there is no doubt that photosynthetic contributions begin as the circinate emerging leaflets (Figure 1c) begin to unfold. Although the sporophyll surfaces of *C. micronesica* male cones are not green, there may be cryptic chlorophyll and photosynthetic activity that has not been verified to date. More gas exchange studies are needed to increase our understanding of photosynthesis and respiration traits of cycad stems, leaves, and reproductive structures. Second, the greater decline in stem NSCs following leaf construction compared with following cone construction may have been a function of the relative duration of the ephemeral sink activity. The entire leaf expansion process for *C. micronesica* culminates in about one month, yet the male cone expansion is distributed over about two months [10]. This doubling of the duration of sink activity for cone construction may reduce the daily sink demands such that whole-plant labile resources can be mobilized and accessed by the plant to adequately support daily growth. The greater daily sink demands that support leaf construction may rely more heavily on stem resources within the stem tissues close to the apex. More studies are needed to determine the NSC relations of root and basal stem tissues in response to ephemeral sink demands. Third, thermogenic activity is a crucial component of male cone behavior for this and other cycad species [13,14], and this thermogenesis would require non-structural resources that would not be required by leaf construction. The substrate source for cone construction costs may not be the same as the source for cone thermogenesis costs. Manipulative studies may be able to tease apart the NSC relations that support cone construction versus cone thermogenesis.

## Figures and Tables

**Table 1:** Experimental details for studying stem carbohydrate relations of *Cycas micronesica* trees. during male cone or leaf growth. Mean ± standard error, n = 6.

Variable	Cone Flush Defoliated	Cone Flush Control	Leaf Flush Defoliated	Leaf Flush Control
Stem height (m)	2.29 ± 0.03	2.33 ± 0.04	2.38 ± 0.04	2.42 ± 0.02
Base diameter (cm)	25.8 ± 1.6	25.1 ± 1.5	25.9 ± 1.6	26.2 ± 1.3
Pith diameter (cm)	5.0 ± 0.5	5.2 ± 0.4	5.3 ± 0.3	5.2 ± 0.4
Vascular diameter (cm)	2.0 ± 0.2	2.1 ± 0.1	2.1 ± 0.1	2.1 ± 0.2
Cortex diameter (cm)	3.1 ± 0.3	3.2 ± 0.2	3.2 ± 0.2	3.1 ± 0.1
